# Nutritional Modulation of Periodontal Diseases: A Narrative Review of Recent Evidence

**DOI:** 10.7759/cureus.50200

**Published:** 2023-12-08

**Authors:** Bandar M Barnawi, Nada S Alrashidi, AlBandari M Albalawi, Nouf S Alakeel, Jmeela T Hamed, Afnan A Barashid, Mohammed S Alduraibi, Ghadeer S Alhussain, Jalal Y Alghadeer, Naser A Alarifi, Abdulaziz M Altalhi

**Affiliations:** 1 Dentistry, Primary Health Care, Madinah, SAU; 2 Dentistry, Security Force Hospital, Riyadh, SAU; 3 Dentistry, Alfarabi Medical Collage, Jeddah, SAU; 4 College of Dentistry, King Saud University, Riyadh, SAU; 5 General Dentistry, Al Mikwa Hospital, Al Baha, SAU; 6 Radiology, Maternity and Children Specialized Hospital, Jeddah, SAU; 7 Dentistry, Ministry of Health, Riyadh, SAU; 8 Dental, Swan Medical Center, Qassim, SAU; 9 Dentistry, Ministry of Health, Al-Ahsa, SAU; 10 Dental Department, Albaha University, Albaha, SAU

**Keywords:** probiotics, melatonin, vitamins, esstional fatty acids, periodontitis

## Abstract

The role of nutrition in managing periodontal diseases is a dynamic and evolving area of study. This review presents an in-depth analysis of various nutritional elements, including essential fatty acids, proteins, vitamins (D, E, and C), coenzyme Q10, melatonin, and probiotics, and their impact on periodontal health. It synthesizes findings from randomized clinical trials and observational studies to highlight the multifaceted influence of these nutrients on periodontal disease management. Key areas of focus include their role in reducing inflammation, altering the composition of the oral microbiota, and enhancing tissue repair and bone health. The review consistently points to the potential benefits of these nutrients, either as standalone agents or in conjunction with standard periodontal treatments, offering valuable insights for both clinicians and researchers. It advocates for a more nutritionally informed approach to periodontal disease management, emphasizing the importance of a well-rounded, preventive, and therapeutic strategy in dental health.

## Introduction and background

Periodontitis is a complex inflammatory condition that leads to the deterioration of both the hard and soft tissues supporting teeth, primarily due to an imbalance in plaque biofilms [1]. This imbalance triggers immune-inflammatory responses, resulting in the degradation of bone and connective tissue [1]. Clinically, periodontitis manifests as attachment loss, periodontal pockets, bleeding gingiva, and bone loss. These manifestations are visible on radiographs and can potentially lead to tooth loss [1,2]. The World Health Organization's Global Oral Health Status Report 2022 indicates that severe periodontal diseases affect approximately 19% of the global adult population, equivalent to nearly 1 billion individuals [3].

Recent research suggests that dietary habits and low intake of specific vitamins and minerals are directly related to periodontal diseases [4]. Nutrients absorbed from the diet impact tooth development and an individual’s susceptibility to periodontal diseases. Additionally, dietary factors can influence the virulence of specific microbes and the formation of dental plaque and may alter the composition and acidity of saliva, thus contributing to the risk of periodontitis [4,5].

The European Federation of Periodontology's European Workshop on Periodontology (Perio Workshop) has reported promising evidence suggesting that nutritional modulation of periodontal inflammation could serve as an effective preventive or treatment approach in managing periodontitis. Moreover, adjusting levels of metabolic cofactors and nutrients has been observed to reduce inflammatory processes in periodontal diseases. Inadequate nutrient levels may impair the body's ability to combat bacterial infections, highlighting the importance of nutritional supplements and superfoods in lowering the risk of periodontitis [4-6].

In the context of periodontal health, understanding the roles of both micronutrients (such as vitamins) and macronutrients (like proteins and fats) is crucial. While micronutrients, required in smaller quantities, play vital roles in maintaining immune function and reducing inflammatory responses, macronutrients provide the essential energy and structural components necessary for overall health. For instance, vitamins C and D are known for their roles in gingival health and bone mineralization, respectively. The global market for dietary supplements, which includes both micro and macronutrients, has experienced significant growth, reflecting heightened public awareness and interest in nutritional supplementation. This trend is partly driven by the growing body of research linking diet to various health conditions, including periodontal diseases. Moreover, the influence of dietary patterns on the oral microbiome and consequently on dental plaque formation and composition underscores the intricate relationship between nutrition and oral health. As such, nutritional counseling and interventions are increasingly relevant in periodontal therapy, paving the way for holistic approaches to dental care [7,8].

This review aims to critically assess the influence of various nutritional supplements administered systemically via the oral route on periodontal health. This review seeks to clarify their potential roles and effectiveness as adjunctive treatments in managing periodontal diseases. By concentrating on nutritional supplementation, it offers a comprehensive overview for dental professionals and researchers, focusing on the systemic effects of these nutrients in periodontal therapy.

## Review

Research methodology

We conducted an extensive electronic database search to gather scholarly articles on the interplay between various nutritional elements and periodontal health. The databases accessed included PubMed, Embase, MEDLINE, and Google Scholar. The search strategy employed a combination of keywords tailored to the focus areas of the review, encompassing: "Essential Fatty Acids and Periodontitis," "Protein and Amino Acid Supplements and Periodontitis," "Vitamin D and Periodontitis," "Vitamin E and Periodontitis," "Vitamin C and Periodontitis," "Coenzyme Q10 and Periodontitis," "Melatonin and Periodontitis," "Probiotics and Periodontitis," and other relevant variations. The inclusion criteria for the literature search were as follows: the articles had to be published in English and be full-text research articles. The scope of the study types was broad, including prospective or retrospective studies, randomized controlled trials, case series, case reports, review papers, and books. These resources were selected to provide a comprehensive understanding of the relationship between dietary supplements, macro- and micronutrients, and periodontal health. The exclusion criteria set for this research were: studies published in any language other than English; research with outcomes not directly relevant to the intersection of nutritional factors and periodontitis; and studies that did not focus on the specific nutritional elements of interest in the context of periodontal diseases.

In response to the diverse nature of research in this field, we extended the time frame of our literature search to include seminal works as well as the most recent studies, ensuring comprehensive coverage of the topic. While recent studies were prioritized, especially those published in the last five years, older but highly influential studies were also considered to provide a thorough historical context and foundational understanding. This approach was chosen to maintain the focus of the manuscript on the synthesis of findings, balancing the depth of analysis with practical considerations in the scope of the literature review. Table 1 shows the articles involved in the comparative review.

**Table 1:** Summary of articles in comparative review

Author(s)	Type of study	Year	Food supplement	Intervention details	Findings	Duration
Stańdo-Retecka et al. [9]	Randomized clinical trial	2023	Essential fatty acids	The test group received scaling and root planning along with a daily oral dose of 20 ml containing omega-3 PUFAs and vitamins A and D3. Clinical assessments at 3 and 6 months included probing depths, attachment levels, bleeding, and closed pocket rates. Bacterial counts and serum lipid profiles were analyzed at baseline, 3 months, and 6 months.	Results showed significant clinical improvements at 3 and 6 months in both groups. The omega-3 group had lower bleeding rates and higher attachment gains at 3 months. By 6 months, differences between the groups were minimal, except in bleeding rates. The omega-3 group also showed a reduced count of periodontal bacteria and altered serum fatty acid proportions after 6 months.	6 months
Stańdo-Retecka et al. [10]	Randomized clinical trial	2020	Essential fatty acids	In a study on omega-3 PUFA's impact on stage III and IV periodontitis, 30 patients received scaling and root planning. The test group of 16 also took omega-3-rich fish oil (2.6 g EPA and 1.8 g DHA) for three months, while the control group of 14 received only the dental treatment. Their periodontal health and salivary samples were evaluated at baseline and after three months.	The test group showed a significant decrease in bleeding on probing and betterment in clinical attachment loss compared to the control group. They also had more closed pockets (probing depth ≤4 mm without bleeding) and improved cytokine levels, with lower pro-inflammatory IL-8 and IL-17 and higher anti-inflammatory IL-10, after three months of omega-3 PUFA supplementation	3 months
Aral et al. [11]	Cross-sectional comparative study	2017	Not applicable	This study compared 25 bodybuilders with gingivitis who used protein supplements (BB-G) to 25 non-exercising males with (G) and 25 without gingivitis (H). It analyzed saliva, gingival fluid, and serum for gene expression of IL-1β, ASC, and CASP1. Periodontal health was assessed through various indices including plaque index (PI), gingival index (GI), and bleeding on probing (BOP).	In the group of bodybuilders using protein supplements, there was a notable reduction in inflammation markers in both gingival fluid and saliva. This suggests that bodybuilding combined with protein supplements might help in reducing gingival inflammation.	Not applicable
Fawaz et al. [12]	Cross-sectional comparative study	2020	Not applicable	The study compared 55 male bodybuilders, aged 35-44, who exercised regularly and used similar protein supplements, with non-bodybuilder peers. They were recruited from fitness centers and their periodontal health was assessed using the Community Periodontal Index and attachment loss.	Bodybuilders showed significantly lower Community Periodontal Index scores compared to non-bodybuilders, indicating better periodontal health. The occurrence of periodontal pockets measuring 4–5 mm was substantially lower in bodybuilders (49.1%) than in non-bodybuilders (80.7%)	Not applicable
Adegboye et al. [13]	Danish Health Examination Survey	2015	Not applicable	A questionnaire was administered to evaluate dietary intakes, focusing on casein, whey proteins, vitamin D, and calcium.	The study found that higher intakes of calcium, casein, and whey protein were associated with a lower risk of periodontitis.	2007-2008
Lee et al. [14]	Randomized control study	2014	Protein (supplement)	14 patients were randomly chosen to consume nutritional supplement drinks for 8 weeks, while a control group of 9 patients did not receive these supplements. Gingival index and tooth mobility were measured at the start, and then at 1, 4, and 8 weeks	The gingival index in the supplement-consuming group showed a significant reduction after just 1 week, unlike the control group. However, by 8 weeks, there was no significant difference in gingival index between the two groups. Tooth mobility also showed significant changes.	8 weeks
Abreu et al. [15]	Cross-sectional study	2016	Not applicable	The study involved 24 participants with moderate to severe periodontitis and 24 periodontally healthy controls, aged 35 to 64 years. Each participant completed a socio-demographic questionnaire, underwent a full-mouth periodontal examination, and provided a blood sample for measuring serum 25-hydroxyvitamin D levels to assess vitamin D status.	The study found that serum 25-hydroxyvitamin D levels were significantly lower in the group with periodontitis compared to the healthy control group.	Not applicable
Isola et al. [16]	Cross-sectional study	2020	Not applicable	The study included 46 patients with chronic periodontitis, 45 with coronary heart disease, 45 with both conditions and 43 healthy individuals. It assessed serum levels of 25(OH)vitamin D and measured clinical attachment, probing depth, and bleeding on probing.	Patients with chronic periodontitis, and those with both periodontitis and coronary heart disease, had significantly lower serum 25(OH)vitamin D levels compared to those with only coronary heart disease and healthy controls. A decrease in 25(OH)vitamin D levels corresponded with worsening periodontal parameter Conversely, higher vitamin D levels were associated with a greater number of teeth.	Not applicable
Dhulipalla et al. [17]	Cross-sectional study	2023	Not applicable	The study involved 64 participants, split evenly between those with chronic periodontitis and healthy individuals, comprising 33 men and 31 women. TNF-α and vitamin D levels were measured. Clinical measurements included attachment levels, probing pocket depth, gingival bleeding, and plaque indices.	Lower levels of vitamin D were associated with worse periodontal health indicators. Additionally, there was a significant increase in serum levels of the pro-inflammatory cytokine TNF-α in participants with decreased vitamin D levels.	Not applicable
Meghil et al. [18]	Pilot study	2019	Oral vitamin D	In the study, 23 patients with moderate to severe periodontitis underwent intensive single-visit scaling and root planning. They were then divided into two groups: one received 4,000 IU/day of oral vitamin D supplementation for 16 weeks (test group), and the other received a placebo.	The test group, which received vitamin D supplementation, showed a reduction in peripheral blood CD3 and CD3+CD8+ cytotoxic T lymphocytes, as well as decreased pro-inflammatory salivary cytokines. Additionally, this group exhibited higher levels of autophagy-related proteins and other proteins essential for anti-microbial autophagy in their whole blood PBMCs (peripheral blood mononuclear cells).	16 weeks
Perić et al. [19]	Randomized double-blind placebo-controlled study	2020	Oral vitamin D	The study involved 13 Caucasian patients with periodontitis and low serum 25(OH) vitamin D3 levels (below 30 ng/mL). They were assigned to a test group that received both scaling and root planning and 25,000 international units (IU) of vitamin D per week.	There was an improvement in clinical parameters, notably in the reduction of pocket depth among patients in the test group, though the differences were not statistically significant.	6 months
Hans et al. [20]	Cross-sectional study	2023	Not applicable	100 subjects, 50 with generalized chronic periodontitis and 50 healthy. Periodontal clinical measurements and serum analysis for various micronutrients.	Lower levels of micronutrients including vitamin E in periodontitis group but not significant.	Not applicable
Behfarnia et al. [21]	Clinical trial	2021	Oral vitamin E	16 chronic periodontitis patients were divided into two groups. Clinical indices measurement and saliva collection pre- and post-treatment with scaling and root planning. The test group took 200 IU vitamin E daily for 2 months.	There were no significant differences in antioxidant capacity and pocket depth changes between the groups. However, the test group showed significantly less attachment loss compared to the control group.	2 months
Assaf and Rabi [22]	Cross-sectional study	2022	Not applicable	The study involved collecting serum samples from 25 patients to evaluate vitamin C deficiency in those with periodontal diseases. Serum vitamin C levels were measured, and the stages and grades of periodontitis were assessed.	Patients with stage IV periodontitis had significantly lower vitamin C levels compared to those in the earlier stages of the disease. However, no notable differences in vitamin C levels were observed between the other stages of periodontitis	November 2020-2021
Munday et al. [23]	Cross-sectional study	2020	Not applicable	20 periodontitis patients, regardless of the stage, and included a periodontal assessment and care, followed by a dietary analysis and measurement of serum vitamin C and C-reactive protein (CRP) levels.	Lower vitamin C levels were linked to more advanced stages of periodontal disease. Two-thirds of the participants with low vitamin C also had elevated CRP levels, indicating a negative correlation between vitamin C and CRP. a higher consumption of processed meats was associated with lower vitamin C levels.	Not applicable
Ghasemi et al. [24]	Randomized control study	2022	Oral coenzyme Q10	42 diabetic patients with chronic periodontitis were split into two groups: 21 received scaling and root planning plus coenzyme Q10, and 21 received only scaling and root planning. Their periodontal health, including attachment level, probing depth, bleeding, gingival index, and plaque index, was evaluated at baseline and after 30 days.	One-month post-intervention, the group receiving Co Q10 supplementation showed significantly lower PPD, CAL, BOP, and PI scores compared to the control group. The GI scores were similar in both groups after the intervention, but both groups experienced a significant decrease in GI scores.	1 month
Shoukheba and El-Kholy [25]	Randomized control study	2019	Oral coenzyme Q10	30 chronic periodontitis patients with type II DM were divided into control (SRP + placebo) and test (SRP + coenzyme Q10) groups. Measurements taken at various intervals included BOP, GI, PPD, CAL, and GCF-MMP-8 levels.	There were statistically significant differences in favor of the test group at all evaluation periods. In the test group, GCF-MMP-8 levels significantly decreased from baseline to 3 months and then slightly increased at 6 months, remaining below baseline values for both groups	3 months, study end at 6 months
Balaji et al. [26]	Cross-sectional study	2015	Not applicable	Five healthy individuals and 15 chronic periodontitis patients were provided 5 ml of saliva, 2 ml of blood, and gingival tissue samples in a fasting state for melatonin analysis.	Melatonin levels in gingival tissue were significantly lower in patients with chronic periodontitis compared to healthy individuals.	Not applicable
Ghallab et al. [27]	Pilot study	2016	Not applicable	65 subjects: 15 healthy individuals, 25 with chronic periodontitis, and 25 with generalized aggressive periodontitis. The levels of malondialdehyde, superoxide dismutase, and melatonin in the GCF were analyzed using enzyme-linked immunosorbent assay.	In the control group, malondialdehyde levels were significantly lower compared to both periodontitis groups. Conversely, superoxide dismutase and melatonin levels were higher in the control group than in the periodontitis groups.	March 2013 to December 2013
El-Sharkawy et al. [28]	Randomized control study	2019	Oral melatonin	In a 6-month trial, 74 chronic periodontitis patients with insomnia were divided into a melatonin group (38 patients) receiving scaling, root planning, and nightly 10 mg melatonin, and a control group (36 patients) receiving scaling, root planning, and a placebo.	The melatonin group showed more significant improvements in clinical attachment levels and pocket depth reductions than the control group after 3 and 6 months. Additionally, their salivary TNF-α levels and insomnia scores were lower. Both groups had similar improvements in bleeding on probing.	2-month regimen, and study end at 6 months
Anton et al. [29]	Randomized control study	2021	Oral melatonin	54 periodontitis and diabetes patients were randomly divided into a test group (27 subjects with scaling and root planning plus melatonin) and a control group (27 subjects with scaling and root planning plus placebo). Periodontal health and glycated hemoglobin (HbA1c) levels were evaluated at the start and 8 weeks later.	The test group exhibited statistically significant improvements in periodontal health and a notable reduction in glycated hemoglobin levels compared to the control group.	8 weeks
Zare Javid et al. [30]	Randomized control study	2020	Oral melatonin	50 type 2 diabetic patients with periodontal disease were split into two groups. The test group received 250 mg/day of melatonin (2 tablets), and the control group received a placebo. All patients underwent scaling and root planning at the study's start. Serum levels of interleukin-1b, malondialdehyde, total antioxidant capacity, superoxide dismutase, catalase, and glutathione peroxidase were measured before and after the intervention	The test group exhibited significantly higher serum levels of total antioxidant capacity, superoxide dismutase, catalase, and glutathione peroxidase. In contrast, their serum levels of interleukin-1b and malondialdehyde were significantly lower compared to the control group.	8 weeks
Invernici et al. [31]	Randomized clinical trials	2018	Bifidobacterium lactis HN019 lozenges	41 chronic periodontitis patients were split into two groups: 20 in the test group received scaling and root planning plus probiotics for 30 days, and 21 in the control group received scaling and root planning plus placebo. Their periodontal health was monitored before, and 30 and 90 days after treatment.	The test group showed significantly fewer periodontal pathogens and lower proinflammatory cytokine levels than the control group. Additionally, the test group had a greater decrease in probing pocket depth and higher clinical attachment gain at 90 days compared to the control group.	30 days, and study end at 90 days
Ghazal et al. [32]	Randomized clinical trials	2023	*Lactobacillus reuteri* tablets	60 smokers with stage III, grade C generalized periodontitis in two groups. Group 1 (SRP + antibiotics + placebo for probiotics) and group 2 (SRP + *L. reuteri* + placebo antibiotics). Periodontal parameters were recorded at baseline, 1, and 3 months.	Both groups showed significant clinical improvement, but differences between the groups were not statistically significant for periodontal parameters.	90 days
Szkaradkiewicz et al. [33]	Randomized clinical trials	2014	*L. reuteri* tablets	38 patients with moderate chronic periodontitis, 24 received probiotic tablets containing *L. reuteri* post-teeth cleaning, while 14 did not (control group). Their gingival crevicular fluid was tested for pro-inflammatory cytokines.	Patients treated with *L. reuteri* showed significant reductions in pro-inflammatory cytokines and improved periodontal health, unlike the control group, indicating the probiotic's effectiveness in reducing periodontal inflammation.	Not specified
Riccia et al. [34]	Double-blind paired-comparison study	2007	*L. brevis*-containing lozenges	Eight healthy individuals and 21 chronic periodontitis patients were treated with *L. brevis* lozenges. Their periodontal health was assessed and saliva samples were analyzed for various markers before and after treatment.	Post-treatment, all patients showed significant improvements in periodontitis symptoms, accompanied by reduced levels of nitrite/nitrate, prostaglandin E2, matrix metalloproteinase, and gamma-interferon in saliva.	Not specified

Essential fatty acids

While exploring the intricate relationship between essential fatty acids and periodontal health, it is crucial to understand the foundation of omega-3 and omega-6 fatty acids. Alpha-linolenic acid (ALA) anchors the omega-3 family, while linoleic acid forms the cornerstone of omega-6. Fundamental research unveils the pivotal role of omega-3 derivatives, specifically docosahexaenoic acid (DHA) and eicosapentaenoic acid (EPA), in benefiting the human body. These compounds are particularly noted for their robust anti-inflammatory properties. The anti-inflammatory action of these fatty acids involves their incorporation into cell membranes, where they compete with omega-6 fatty acids for the same metabolic enzymes, leading to the production of different eicosanoids. The eicosanoids derived from omega-3s are generally less inflammatory than those derived from omega-6s, thereby reducing the overall inflammatory response in the body [35].

Furthermore, omega-3 fatty acids contribute to the production of specialized pro-resolving mediators (SPMs) like resolvins, protectins, and maresins, which play a crucial role in the resolution of inflammation. This process is especially important in tissues affected by periodontal diseases, where unresolved inflammation can lead to tissue damage.

These essential fatty acids are available in convenient forms, such as tablets and oils found in pharmacies, serving as accessible food supplements. Popular sources include fish oil capsules, which are rich in EPA and DHA, and plant-based alternatives like flaxseed oil or algal oil supplements, which are high in omega-3s. Incorporating these supplements into one's diet, typically in doses ranging from 0.5 to 1.5 g, may contribute to maintaining cell membrane fluidity. This is crucial since the fatty acid composition of cell membranes is dynamic and reflective of dietary intake. Regular consumption of these supplements ensures that cell membranes remain fluid and functional, enhancing their ability to respond to various cellular signals and reducing the release of proinflammatory cytokines by monocytes/macrophages [35].

Upon ingestion, these fatty acids are absorbed in the small intestine and transported to various tissues via the bloodstream. Adipose tissue stores excess fatty acids and can mobilize them as needed. Eventually, the body excretes the metabolites and byproducts of fatty acid metabolism, completing their lifecycle [35]. This process underscores the necessity of consistent intake to sustain the beneficial effects of these fatty acids, particularly in the context of periodontal health, where inflammation plays a central role.

In a recent randomized clinical trial conducted by Stańdo-Retecka et al., the test group, comprised of patients diagnosed with generalized periodontitis stage III or IV, received high doses of omega-3 polyunsaturated fatty acids (PUFAs) - specifically, 2.6 g of EPA and 1.8 g of DHA - over a short-term period of six months, resulting in notable improvements in the resolution of inflammation, reflected in reduced pocket depth and bleeding on probing [9]. On a six-month follow-up, the test group exhibited consistently lower levels of specific bacteria, including Porphyromonas gingivalis, Tannerella forsythia, Treponema denticola, and Aggregatibacter actinomycetemcomitans, in comparison to the control group [9]. This finding underscores the potential of omega-3 PUFA supplementation to influence the oral microbiota. However, it is noteworthy that Stańdo-Retecka et al. reported no significant differences between the control and test groups after six months regarding certain clinical parameters [9]. This observation suggests that the initial positive effects of omega-3 PUFA supplementation on periodontal health may not have been sustained over a more extended period [9]. Adding to the evidence from the work by Stańdo-Retecka et al., a previous publication by the same author revealed that patients supplemented with omega-3 PUFAs exhibited marked reductions in the levels of pro-inflammatory cytokines/chemokines interleukin (IL)-8 and IL-17. Concurrently, the anti-inflammatory IL-10 demonstrated a significant increase in salivary samples [10]. These findings provide additional context for the potential mechanisms through which omega-3 PUFAs may impact inflammatory processes in the oral environment, shedding light on the intricate relationship between supplementation and cytokine modulation. Building upon this evidence, a preceding publication by Castro Dos Santos further bolsters the case. His systematic review and meta-analysis aimed to assess the impact of dietary supplements of omega-3 fatty acids as an adjunct to non-surgical periodontal treatment, in comparison with periodontal treatment alone, on the periodontal clinical parameters of patients diagnosed with periodontitis. The outcomes revealed a statistically significant and consistent overall reduction in pocket probing depth (PPD) by 0.42 mm and a gain in clinical attachment level (CAL) of 0.58 mm [36]. These findings collectively suggest a promising positive effect of incorporating dietary omega-3 fatty acids into the treatment regimen for periodontitis patients, indicating enhanced periodontal clinical parameters compared to traditional periodontal treatment alone. The harmonious alignment of outcomes from both studies implies a consistent and synergistic impact between omega-3 fatty acids and non-surgical periodontal treatment, emphasizing their potential to elevate certain periodontal health parameters [36].

The clinical improvements observed at the three-month mark in the study by Stańdo-Retecka et al. can be attributed to the increased levels of omega-3 fatty acids due to supplementation, which likely induced a strong, immediate anti-inflammatory response. This is reflected in the reduced pocket depth and bleeding [9]. However, the diminished impact observed at the six-month follow-up, particularly in certain clinical parameters, suggests a potential decline in the efficacy of these fatty acids over time, possibly due to their metabolic turnover and concentration in the body [9].

In analyzing the study by Stańdo-Retecka et al., the duration of omega-3 PUFA supplementation is a key factor. The supplementation ended at three months, with observations extending to six months [9]. Continuing supplementation until the study's endpoint could have offered a clearer picture of the long-term effects on periodontal health. Extending the supplementation period would help determine if the initial improvements are sustained or if discontinuing the omega-3 fatty acids at six months is responsible for the reduced efficacy. A longer supplementation period in future studies could provide deeper insights into the lasting benefits and safety of omega-3 PUFAs in periodontal therapy, informing more effective treatment protocols.

It is critical to consider the uniformity often assumed in clinical studies regarding participants' diets and lifestyles. Such an assumption overlooks individual variations that significantly affect the metabolism and lifecycle of fatty acids. Personal dietary habits, physical activity levels, and metabolic rates can greatly influence the processing and utilization of these fatty acids, leading to varied responses to supplementation. This individual variability might explain the differences in results seen across studies and over time within the same study.

Therefore, while omega-3 fatty acids demonstrate potential in periodontal therapy, their effectiveness hinges not only on the dosage but also on the duration of supplementation, individual metabolic responses, and lifestyle factors. The results from different studies [9,10,36], when viewed in this broader context, highlight the necessity of a personalized approach to omega-3 fatty acid supplementation. This approach should account for individual dietary patterns, lifestyle choices, and metabolic differences. Future research in this area should aim to better understand these dynamics and how they influence the long-term effectiveness of omega-3 fatty acids in managing periodontal health and other clinical conditions.

Protein and amino acid supplements

Recently, there has been a growing trend in the use of fitness supplements, with protein supplements, particularly whey protein, being the most popular choice among fitness enthusiasts for muscle growth and strength enhancement. Proteins play a crucial role not only as essential structural and functional components of the skeletal system but also in promoting overall health, including periodontal wellness. Inadequate protein intake has been linked to delayed wound healing in humans [7], underscoring the importance of adequate protein in the diet.

Whey protein, a widely used protein supplement, is especially notable for its rich content of essential amino acids, including leucine, isoleucine, and valine, which are vital for muscle repair and growth. Beyond its muscle-building benefits, whey protein also exhibits anti-inflammatory and antioxidant properties. It influences cytokine production, balancing pro-inflammatory and anti-inflammatory responses in the body. This modulation of the immune system is particularly relevant in managing inflammation, which is a key factor in periodontal health and wound healing. Additionally, whey protein's antioxidant capabilities, primarily due to its cysteine content, which boosts glutathione levels, help combat oxidative stress. This reduction in oxidative stress is beneficial for protecting cells from damage and supporting overall bodily functions.

Therefore, the consumption of whey protein extends beyond its traditional role in muscle development. Its impact on immune modulation and antioxidant defenses makes it a valuable dietary component, not only for fitness enthusiasts but also for individuals looking to support their general health, including the maintenance of healthy periodontal tissues. As always, it is important to balance whey protein intake as part of a well-rounded diet and consult healthcare professionals for personalized dietary advice, especially in the context of specific health conditions [7,11-14].

In their study, Aral et al. investigated the impact of bodybuilding and protein supplements on periodontal tissue. The test group, composed of bodybuilders with gingivitis, exhibited no significant distinctions in clinical parameters compared to other groups. However, the results suggested that the combination of bodybuilding activities and supplement usage might lead to a decrease in gingival inflammation by downregulating caspase-1 (CASP1), interleukin-1β (IL-1β), and apoptosis-associated Speck-like protein containing a CARD (ASC) [11].

Similarly, an observational comparative study by Fawaz and colleagues in 2020 delved into the relationship between the periodontal status of male bodybuilders and their intake of protein supplements. The findings highlighted a higher prevalence of periodontal pocket depth (4-5 mm) in 80.7% of non-bodybuilders compared to 49.1% of bodybuilders who consumed protein supplements [12]. In a cross-sectional study led by Adegboye et al., a consistent inverse relationship was observed between whey protein intake and periodontitis [13]. Furthermore, Lee et al. examined the effects of nutritional supplements (protein, vitamin A, B1, and niacin) on post-surgical periodontal health and tooth mobility. Their eight-week study after periodontal surgery demonstrated that regular intake of these supplements significantly improved periodontal recovery, as indicated by enhanced plaque index (PI), gingival index (GI), and reduced tooth mobility [14].

More controlled studies are needed to understand the effects of protein supplements on periodontal health. Bodybuilders, due to their unique dietary and recovery patterns, may not represent the standard population. They often consume protein from various sources, and their recovery mechanisms might differ significantly from those of non-athletes. Additionally, standardizing the brand and type of protein supplements used in such studies is crucial for consistency and comparability of results. These studies hint at important considerations for healthcare professionals treating bodybuilders or individuals on high-protein diets. When devising treatment plans or scheduling follow-up visits, the potential impact of protein supplementation on periodontal health should be considered.

Vitamins

In the realm of healthcare, the role of vitamins emerges as a crucial facet in maintaining optimal bodily functions. These micronutrients, inherently linked to our dietary intake, serve as indispensable regulators and catalysts for a myriad of physiological processes. Within this context, our exploration focuses on two main categories of vitamins: the water-soluble vitamins, such as B-complex vitamins and vitamin C (which are either absorbed or expelled from the body), and the fat-soluble vitamins, such as vitamins D and E (both stored in the liver and fat tissues). Understanding the intricate interplay between vitamins and overall health is fundamental for oral healthcare practitioners, as it unveils the potential implications for dental well-being and underscores the significance of a comprehensive approach to patient care. Notably, the water-soluble B-complex vitamins and vitamin C contribute to the vitality of oral tissues, supporting the health of gingiva and mucous membranes. Meanwhile, fat-soluble vitamin D plays a pivotal role in calcium absorption and bone metabolism, crucial for the maintenance of a healthy skeletal structure, including teeth. This exploration not only enriches our understanding of the intricate relationship between vitamins and oral health but also emphasizes the pivotal role of these micronutrients in fostering comprehensive patient well-being [7,37].

Vitamin D

Research has consistently affirmed the relationship between vitamin D levels and periodontitis. A pilot study by Abreu et al. in Puerto Rican adults revealed a notable association, with periodontitis being more prevalent among individuals with low serum vitamin D levels [15]. A cross-sectional study conducted by Isola et al. corroborated this finding, demonstrating lower serum vitamin D levels in individuals with periodontitis, as well as those with both periodontitis and coronary heart disease, compared to individuals with only coronary heart disease and healthy controls [16].

In a more detailed examination, Dhulipalla et al. conducted a 2023 study assessing serum levels of 1, 25-dihydroxycholecalciferol (1, 25(OH)2D) and tumor necrosis factor-α (TNF-α) in individuals with chronic periodontitis. The results indicated an inverse relationship between 1,25(OH)2D levels and periodontal parameters. Lower 1,25(OH)2D levels were associated with increased pro-inflammatory cytokines, notably TNF-α [17]. Further supporting this, a pilot study in 2019 aimed to explore the impact of vitamin D supplements on inflammatory markers in periodontitis patients. In this study, 23 patients were randomly assigned to either the vitamin D (4000 IU/day orally) group or the placebo group. Both groups underwent single-sitting scaling and root planning. The findings demonstrated that the administration of vitamin D supplements led to an increase in serum levels of 25-hydroxy vitamin D. Moreover, it resulted in a reduction in blood CD3 and cytotoxic T lymphocyte cells, along with a decrease in proinflammatory salivary cytokines, ultimately contributing to a reduction in inflammation in periodontal tissues [18].

Building on these insights, Perić et al. conducted a randomized clinical trial with a six-month follow-up, focusing on patients with periodontitis and low serum vitamin D3 levels. The test group, receiving weekly vitamin D supplementation, displayed improved clinical parameters, particularly a reduction in pocket depth. Although the differences were not statistically significant, these findings contribute to the growing evidence of the potential benefits of vitamin D in periodontal care [19]. Considering the confirmed association, there is emerging interest in incorporating vitamin D as an adjunct to main periodontal treatments. The studies suggest that optimizing vitamin D levels may complement traditional therapies, potentially enhancing overall treatment outcomes. This connection between vitamin D and periodontitis opens avenues for further exploration, emphasizing a holistic approach to oral health care that integrates nutritional considerations with established treatment protocols.

Recent studies have increasingly highlighted vitamin D as a significant risk factor in the development and progression of periodontitis. This growing body of evidence underscores the potential role of vitamin D, not just as a supplementary treatment but as a crucial preventative measure. However, a key challenge lies in determining the optimal dosage for individual patients. There is notable variation among experts regarding the standard ratio for vitamin D supplementation, with differing recommendations on daily, weekly, or monthly dosages. This variation necessitates a more tailored approach to supplementation, considering individual patient needs, including factors like baseline vitamin D levels, sun exposure, dietary intake, and specific health conditions.

Furthermore, future research in this area should not only focus on clinical outcomes but also incorporate patient education as a core component. Educating patients about the importance of vitamin D for periodontal health, along with practical guidance on sun exposure and dietary sources, could significantly enhance treatment effectiveness. This educational aspect is vital in empowering patients to make informed decisions about their oral health care, especially in understanding how lifestyle factors such as sun exposure can naturally augment their vitamin D levels.

Incorporating these elements into periodontal treatment plans could lead to more personalized and effective care strategies. By embracing a holistic approach that combines clinical treatment with lifestyle and nutritional education, there is potential to significantly improve outcomes for patients with periodontitis. This approach would also align with the broader trend in healthcare towards personalized and preventive medicine.

Vitamin E

A fat-soluble nutrient stands out for its potent antioxidant properties. Renowned for its ability to neutralize free radicals, vitamin E plays a crucial role in protecting cells from oxidative stress. Its functions extend beyond mere antioxidant capabilities; vitamin E is integral to immune function, skin health, and neurological processes [7].

In the realm of periodontal health, research on vitamin E is comparatively scarce compared to vitamin D. Despite studies exploring the connection between periodontitis and vitamin E, the volume of research on this specific vitamin is lower than that on vitamin D. The comparatively lower volume of research on vitamin E in periodontal health versus vitamin D can be attributed to several factors. Vitamin D has garnered extensive attention due to its wide-ranging impact on overall health, including bone, immune, and muscle health, and its potential roles in mood and cardiovascular regulation. Public health concerns over vitamin D deficiency, largely due to lifestyle changes leading to reduced sun exposure, have spurred significant research interest. Additionally, emerging interests in Vitamin D's role in chronic diseases and conditions like diabetes, certain cancers, and COVID-19 have further boosted its research prominence. On the other hand, vitamin E, known for its antioxidant properties, tends to have a more niche research focus. Challenges in researching vitamin E's complex interactions and the historical focus and funding priorities favoring vitamin D also contribute to this disparity. While vitamin E's importance, particularly in combating oxidative stress, is recognized, vitamin D's broader health implications and public health relevance have led to a greater volume of research in the context of periodontal health.

Recently, a cross-sectional study by Hans et al. compared 50 individuals with generalized chronic periodontitis to 50 individuals without signs of periodontitis. Serum samples were collected and analyzed for vitamin E levels. The results revealed lower vitamin E levels in the periodontitis group compared to the healthy control group, although the difference was not statistically significant [20].

Behfarnia et al. conducted a study involving 16 patients diagnosed with chronic periodontitis. All participants underwent scaling and root planing (SRP), with the test group receiving a daily supplement of 200 IU of vitamin E for two months. Post-intervention, clinical indices were reassessed, and saline total antioxidant capacity (TAC) was measured. While there were no significant differences in the mean change in TAC and pocket depth between the two groups, the test group exhibited a significantly lower mean change in attachment loss compared to the control group [21]. Further research is essential to comprehensively understand the impact of vitamin E on periodontal well-being. At present, it would be premature to consider vitamin E deficiency as a definitive risk factor for periodontal disease due to the limited and inconclusive nature of current studies.

Vitamin C

L-ascorbic acid, commonly known as vitamin C, is an indispensable nutrient crucial for human health. Its essentiality stems from the human body's inability to synthesize this molecule due to the absence of L-gulono-1,4-lactone oxidase [38]. Vitamin C exerts a profound influence on various metabolic processes, playing a pivotal role in regulating collagen, corticosteroids, neurotransmitter synthesis, iron absorption, and immune system reactions [38]. Notably, it is instrumental in the formation of intermolecular collagen cross-links, thereby strengthening covalent bonds within tropocollagen molecules [39]. This process is essential for the stability of collagen. A deficiency in vitamin C may contribute to the instability of this protein, potentially weakening periodontal ligaments and leading to tooth loss [39].

In addition to its critical role in collagen formation, vitamin C has been reported to have a protective effect on periodontal tissues. Its adequate intake is essential to reducing the risk of periodontal disease. Vitamin C's ability to decrease levels of proinflammatory cytokines plays a significant role in mitigating inflammatory responses within the periodontium. Furthermore, vitamin C is known to reduce bleeding within the gingiva, a symptom often associated with periodontal disease. This property makes it a valuable component in additional therapies for periodontal conditions. Beyond its impact on collagen and gingival bleeding, L-ascorbic acid also supports endothelial cell function, further underscoring its importance in maintaining the integrity of oral tissues [40]. The intricate relationship between vitamin C and periodontal health highlights its significance not only in preventing and managing periodontal disease but also in contributing to the overall health of the oral cavity. Recent cross-sectional studies have provided compelling insights into the relationship between serum vitamin C levels and the progression of periodontal diseases. Assaf and Rabi [22] and Munday et al. [23] conducted assessments that offer a deeper understanding of this correlation. Assaf and Rabi identified a significant variance in vitamin C levels across different stages of periodontitis, highlighting a clear distinction in vitamin C levels between stages I and IV of periodontitis. This suggests a potential progressive decrease in vitamin C levels as periodontal disease advances. Additionally, Munday et al. reinforced this observation by reporting that patients with vitamin C levels below the normal range (40-100 µmol/L) had a higher stage of periodontal disease, indicating a potential link between vitamin C deficiency and the severity of periodontal conditions.

These studies collectively underscore the critical role of vitamin C in periodontal health and disease progression. The observed association between lower serum vitamin C levels and advanced stages of periodontitis raises important questions about the potential benefits of vitamin C supplementation in managing periodontal diseases. However, despite these valuable insights, there remains a notable gap in the literature, particularly regarding specific investigations into how vitamin C supplementation might influence the outcomes of non-surgical periodontal treatments. This gap points to an unexplored area of research that could significantly enhance our understanding of periodontal therapy. It suggests a need for more targeted studies to investigate whether supplementing with vitamin C can provide tangible benefits in conjunction with traditional non-surgical treatments, such as scaling and root planing. Exploring this could potentially reveal vitamin C supplementation as a valuable adjunctive treatment strategy, possibly aiding in better disease management and improved patient outcomes. Such research would not only fill the existing knowledge gap but also offer a more holistic approach to periodontal care, integrating nutritional supplementation with established periodontal treatment protocols.

Coenzyme Q10

Coenzyme Q10 (CoQ10), a pivotal micronutrient synonymous with energy production and cellular defense, has captured the attention of health enthusiasts and researchers alike. As a vitamin-like compound, CoQ10 plays a dual role: fueling the intricate cellular machinery and shielding cells from oxidative damage. Its significance grows with age, as the body's natural production of CoQ10 diminishes [41], necessitating supplementation to maintain optimal levels. Among its myriad benefits, one area is particularly intriguing: its potential impact on oral health, specifically in combating periodontitis.

In a series of randomized clinical trials, Ghasemi et al. [24] and Shoukheba and El-Kholy [25] explored the effects of coenzyme Q10 on chronic periodontitis. Ghasemi et al. divided patients, including those with diabetes, into control and test groups after an initial periodontal clinical evaluation. Both groups underwent scaling and root planning, but the test group also received one capsule of CoQ10 daily for a month. This additional supplementation led to significant improvements in bleeding on probing, clinical attachment level, probing pocket depth, and the plaque index. It is important to note that HbA1C levels, a key measure of blood sugar control over time, were not assessed in this study, which could have provided additional insights into the impact of CoQ10 on periodontal health, especially in patients with diabetes [24]. In a parallel study conducted by Shoukheba and El-Kholy, two capsules of CoQ10 were administered daily to patients with chronic periodontitis, including those with diabetes, for three months. This regimen achieved similar improvements in clinical parameters, as seen in the study by Ghasemi et al. Notably, this study observed a significant reduction in matrix metalloproteinase-8 (MMP-8) levels in the gingival crevicular fluid. The focus on MMP-8 levels is particularly interesting, as MMP-8 may have a correlation with periodontal health in diabetic patients, and its levels can be indicative of diabetic complications, particularly in terms of wound healing [25]. Although diabetic patients were included in this study, it did not assess whether CoQ10 supplementation had a synergistic effect with scaling and root planing, particularly in terms of HbA1c, a key indicator of long-term glycemic control.

While the research on oral supplementation of Coenzyme Q10 for periodontal health shows some positive indications, it remains limited and not as promising as one might hope. The studies conducted by Ghasemi et al. [24] and Shoukheba and El-Kholy [25] suggest potential benefits, but the overall body of research lacks the depth and robust structure necessary for definitive conclusions. Future studies should be more meticulously structured, possibly with larger sample sizes, longer durations, and more comprehensive parameters, including HbA1c levels for diabetic patients, to better assess the efficacy of CoQ10 in periodontal treatment.

Melatonin

Melatonin, a hormone produced by the pineal gland, is widely recognized for more than just its role in regulating sleep. It is a powerful antioxidant with notable anti-inflammatory and immunomodulatory properties. As an antioxidant, melatonin plays a crucial role in defending the body against oxidative stress. It scavenges harmful free radicals, thereby protecting cells from the damage these reactive species can cause. This antioxidant action is vital in mitigating the risk of chronic diseases associated with oxidative stress, such as neurodegenerative disorders, cardiovascular diseases, and aging-related conditions. Additionally, melatonin has significant effects on bone health. It enhances osteogenic gene expression, leading dental pulp stem cells (DPSCs) to differentiate into osteoblasts, which are vital for bone formation. This hormone also plays a key role in maintaining bone density by reducing bone resorption, achieved through the inhibition of osteoclast activity. Further, melatonin supports the osteogenic differentiation of bone marrow stromal cells (BMSCs), contributing to bone formation and preventing bone loss. The comprehensive effects of melatonin, from its antioxidant properties to its impact on bone health, highlight its multifaceted role in human physiology [42]. Understanding these diverse roles of melatonin is crucial, especially when considering its potential impact on periodontal diseases like periodontitis.

In a series of cross-sectional studies, researchers have made significant observations regarding the levels of melatonin in relation to periodontal health. The study by Balaji et al. involved a detailed examination of melatonin levels in patients with chronic periodontitis. The study obtained 5 ml of whole saliva, 2 ml of peripheral blood, and gingival tissue samples from each participant, comprising five healthy individuals and fifteen chronic periodontitis patients. These samples were collected at 8:00 a.m. in a fasting state for an accurate melatonin assay. Notably, it was found that melatonin levels were significantly lower in the gingival tissue of chronic periodontitis patients when compared to healthy individuals [26]. Complementing these findings, Ghallab et al. reported similar results, indicating that melatonin levels were significantly reduced in patients with aggressive periodontitis compared to those with chronic periodontitis [27]. Further cementing these observations, a recent systematic review and meta-analysis by Balaji et al. concluded that individuals with chronic periodontitis exhibited notably lower levels of melatonin in their saliva compared to healthy controls [43]. These studies collectively underscore the potential link between reduced melatonin levels and the severity of periodontal diseases.

In a series of randomized clinical trials, the efficacy of melatonin as an adjunct to periodontal treatment has been explored, with notable findings. El-Sharkawy et al. conducted a six-month trial with 74 patients suffering from generalized chronic periodontitis. The study divided participants into two groups: the test group received standard root planing (SRP) along with a two-month regimen of 10 mg oral melatonin capsules daily before bedtime, while the control group underwent SRP and was given placebo capsules. Results showed that the melatonin group had significantly greater clinical attachment level (CAL) gains, pocket depth (PD) reductions, and lower levels of salivary TNF-α and AIS scores compared to the placebo group [28]. Complementing these findings, Anton et al. conducted a trial including type II diabetic patients. The test group received SRP and 3 mg of melatonin daily for eight weeks. This study observed that the test group had significantly lower bacterial plaque and gingival bleeding indices, along with a notable reduction in HbA1c levels compared to controls [29]. Similarly, Ahmed et al. investigated the antioxidant and anti-inflammatory properties of melatonin in patients with type 2 diabetes mellitus. In this trial, the test group received SRP along with 500 mg of melatonin daily. The findings indicated significant decreases in serum levels of interleukin-1b and malondialdehyde, along with increases in total antioxidant capacity, superoxide dismutase, catalase, and glutathione peroxidase [30]. These trials collectively highlight the potential benefits of incorporating melatonin into periodontal therapy, particularly in enhancing clinical outcomes and reducing inflammatory markers in patients with periodontitis, including those with systemic conditions like diabetes.

The relationship between melatonin levels and periodontal health is becoming increasingly evident, with studies consistently showing that lower levels of melatonin are associated with periodontitis. This correlation underscores the potential role of melatonin in maintaining oral health and preventing periodontal diseases. However, when it comes to the therapeutic use of systemic melatonin in conjunction with scaling and root planning for periodontal treatment, the optimal dosage remains unclear. There is a noticeable variation in the dosages of systemic melatonin used across different studies, indicating a need for standardization and more comprehensive research. This variability highlights the complexity of integrating melatonin into periodontal therapy and underscores the importance of determining the most effective and safe dosage to enhance treatment outcomes.

Probiotics

Probiotics, often referred to as "good bacteria," are gaining increasing recognition in the fields of nutrition and health. These live microorganisms, when consumed in adequate amounts, offer numerous health benefits. They are particularly noted for balancing the body's natural microbiome, which is crucial for overall well-being. Probiotics have shown promise in enhancing digestive health, boosting immune function, and even improving mental health through the gut-brain axis. Researchers are also exploring the role of probiotics in oral health, as evidence suggests they can help maintain a healthy balance of oral bacteria [44]. This is important for preventing the overgrowth of harmful bacteria that can lead to oral diseases.

Recent randomized clinical trials have investigated the efficacy of probiotics in periodontal therapy, with varying results. Invernici et al. conducted a placebo-controlled trial where both test and control groups received scaling and root planning, but the test group additionally took Bifidobacterium lozenges twice daily for 30 days. This group exhibited significantly higher probing pocket depth reduction and clinical attachment gain, along with fewer periodontal pathogens of red and orange complexes and lower proinflammatory cytokine levels compared to the control group [31].

In contrast, the study by Ghazal et al., focusing on smokers with stage III, grade C generalized periodontitis, compared the effects of antibiotics (amoxicillin and metronidazole for seven days) with *Lactobacillus reuteri* probiotics (one tablet twice daily for 30 days) following nonsurgical periodontal treatment. Interestingly, this trial found no significant difference in periodontal parameters between the two groups by the end of the trial. This parity in results between the antibiotic and probiotic groups draws increased attention to the potential of probiotics as an alternative or adjunct to antibiotics in periodontal treatment [32].

Further research by Szkaradkiewicz et al. demonstrated that *L. reuteri* tablets led to significant improvements in the sulcus bleeding index, probing pocket depth, clinical attachment loss, and a reduction in pro-inflammatory cytokines in chronic periodontitis patients [33]. Similarly, a study by Riccia et al. found that *L. brevis* lozenges significantly enhanced the plaque index and gingival index and reduced salivary prostaglandin E2 and matrix metalloproteinases in individuals with periodontitis [34].

In analyzing the limitations of probiotic usage, particularly in the context of periodontal health, several critical factors emerge. First and foremost, the efficacy of probiotics is not a one-size-fits-all solution; it varies greatly depending on individual factors such as the specific strains used, dosages, and the unique gut flora of each person. This variability underscores the complexity of predicting outcomes in periodontal treatments involving probiotics. Additionally, individual differences play a substantial role. The diverse nature of each person's microbiome, genetic background, and health status means that the effectiveness of probiotics can differ markedly from one individual to another, complicating their use in standardized periodontal treatment protocols. Safety concerns add another layer of complexity. The use of probiotics can potentially pose health risks, especially in individuals with compromised immune systems, which must be taken into account in periodontal therapy. Furthermore, the lack of standardization in the production and labeling of probiotics raises questions about the consistency and reliability of these supplements, which is a crucial factor in their therapeutic application. The paucity of long-term research data on probiotics limits our understanding of their sustained impact, especially in the context of chronic conditions like periodontal disease. Lastly, the regulatory landscape for probiotics, often classified as dietary supplements, lacks the stringent testing and approval process mandatory for pharmaceuticals, leading to potential discrepancies in quality and efficacy claims. This analysis highlights the need for a cautious, personalized approach when considering probiotics in periodontal health management, emphasizing the importance of rigorous research and tailored treatment strategies [31-33,44]. Table 2 summarizes the findings.

**Table 2:** Clinical implications of food supplements on periodontal health

Nutritional factor	Type (micro/macronutrient, hormonal/dietary supplement)	Key findings	Implications for periodontal health
Essential fatty acids	Dietary supplement	Omega-3 and omega-6 fatty acids, especially EPA and DHA, show anti-inflammatory properties.	Beneficial in reducing inflammation and improving periodontal health.
Protein and amino acids	Macronutrient/dietary supplement	Whey protein and amino acids like leucine, isoleucine, and valine are linked to improved periodontal status.	May contribute to periodontal tissue repair and health.
Vitamin D	Micro nutrient/dietary supplement	Lower serum vitamin D levels associated with periodontitis; supplementation shows improvement in periodontal parameters.	Crucial for bone health and may aid in periodontal disease management.
Vitamin E	Micronutrient/dietary supplement	Antioxidant properties; studies show mixed results on its impact on periodontal health.	Potential benefits in combating oxidative stress in periodontal tissues.
Vitamin C	Micronutrient	Essential for collagen formation and immune function; deficiency linked to worsened periodontal health.	Important for maintaining gingival health and preventing tooth loss.
Coenzyme Q10	Hormonal substance/dietary supplement	Shown to improve clinical parameters in periodontitis, such as bleeding on probing and pocket depth.	Could be a beneficial supplement in periodontal therapy.
Melatonin	Hormonal substance/dietary supplement	Exhibits anti-inflammatory and bone-strengthening properties; lower levels observed in periodontitis patients.	May play a role in reducing periodontal inflammation and promoting bone health.
Probiotics	Dietary supplement	Probiotics can help balance oral microbiota; studies show varied results in their effectiveness in periodontal treatment.	Potential use as an adjunct to conventional periodontal therapies.

Limitation

This review, while comprehensive, encounters several limitations. These include variable dosages used across studies, which complicates comparisons and conclusions. Patient adherence is challenging due to prolonged supplement intake periods. There's a notable lack of standardization, particularly evident in vitamin D research, regarding optimal levels and deficiency cut-offs. Some nutrients require extended periods to manifest effects on periodontal health, impacting their efficacy in short-term studies. Limited and outdated research on certain supplements restricts the applicability of findings to current practices. Complexities arise when supplements are available in combination formulations, making it difficult to identify the active, beneficial component. Small sample sizes and diverse treatment protocols in clinical studies limit the generalizability of results. There is also an absence of comprehensive guidelines on supplement use for periodontal disease, including considerations of side effects, dosage standardization, and long-term impacts. Moreover, patient-related outcomes and the specific efficacy of increased dietary intake of nutrients like melatonin are underrepresented. Lastly, the complex interactions between different nutritional supplements and conventional periodontal treatments remain underexplored. Table 3 summarizes the limitations of the review.

**Table 3:** Limitations of studies discussed in the review.

Limitation	Description
Variable dosages	Studies reviewed employed a range of dosages for supplements, making comparisons and conclusions challenging.
Patient adherence	The prolonged period required for supplement intake may affect patient adherence, influencing study outcomes.
Lack of standardization	Inconsistencies, especially in vitamin D research, regarding optimal levels and deficiency cut-offs.
Delayed results	Some nutrients may require a long period to show effects on periodontal health, affecting short-term study efficacy.
Limited research on certain supplements	Some popular supplements lack robust studies, while others have limited research, necessitating further investigation.
Outdated research	Absence of recent studies for certain supplements, challenging their application to current clinical practices.
Complex supplements	Difficulty in ascertaining the beneficial component in complex supplement formulations.
Diverse treatment protocols and small sample sizes	Variations in treatment protocols and small sample sizes in studies limit generalizability.
Absence of comprehensive guidelines and consideration of side effects	Lack of detailed guidelines for supplement use in periodontal disease, including dosage standardization, long-term effects, and potential side effects.
Patient-related outcomes and dietary efficacy	Limited coverage of patient-related outcome factors and specific efficacy of increased dietary intake of certain nutrients like melatonin.

## Conclusions

In conclusion, this review has systematically explored the role of various nutritional factors, including essential fatty acids, proteins, vitamins, coenzyme Q10, melatonin, and probiotics, in the modulation of periodontal diseases. The evidence presented underscores the potential of these nutrients to influence periodontal health positively, either through direct effects on oral tissues or by enhancing overall systemic health. However, this review also highlights significant limitations in the current research, such as variable dosages, a lack of standardized protocols, and challenges in patient adherence, which must be considered when interpreting these findings.

Moreover, the underrepresentation of patient-related outcomes and the complex interactions between nutritional supplements and conventional periodontal treatments point to the need for more comprehensive, well-designed clinical trials. Such research would help establish clearer guidelines and understand the long-term effects of these nutritional interventions on periodontal and systemic health. Despite these limitations, the review indicates a promising direction for future research and clinical practice, suggesting that nutritional modulation could be a valuable adjunct to conventional periodontal therapies. Incorporating nutritional strategies into periodontal disease management could lead to more effective, holistic care, improve patient outcomes, and contribute to a better understanding of the intricate relationship between nutrition and oral health.

## References

[1] Listgarten MA (1986). Pathogenesis of periodontitis. J Clin Periodontol.

[2] Murakami S, Mealey BL, Mariotti A, Chapple IL (2018). Dental plaque-induced gingival conditions. J Periodontol.

[3] World Health Organization (2023). Oral health. https://www.who.int/news-room/fact-sheets/detail/oral-health.

[4] Van der Velden U, Kuzmanova D, Chapple IL (2011). Micronutritional approaches to periodontal therapy. J Clin Periodontol.

[5] Tonetti MS, Chapple IL (2011). Biological approaches to the development of novel periodontal therapies--consensus of the Seventh European Workshop on Periodontology. J Clin Periodontol.

[6] Quiles J, Varela-López A (2013). The role of nutrition in periodontal diseases. Studies on Periodontal Disease.

[7] Spahr A, Divnic-Resnik T (2022). Impact of health and lifestyle food supplements on periodontal tissues and health. Periodontol 2000.

[8] Kamiński M, Kręgielska-Narożna M, Bogdański P (2020). Determination of the popularity of dietary supplements using Google search rankings. Nutrients.

[9] Stańdo-Retecka M, Piatek P, Namiecinska M, Bonikowski R, Lewkowicz P, Lewkowicz N (2023). Clinical and microbiological outcomes of subgingival instrumentation supplemented with high-dose omega-3 polyunsaturated fatty acids in periodontal treatment - a randomized clinical trial. BMC Oral Health.

[10] Stańdo M, Piatek P, Namiecinska M, Lewkowicz P, Lewkowicz N (2020). Omega-3 polyunsaturated fatty acids EPA and DHA as an adjunct to non-surgical treatment of periodontitis: a randomized clinical trial. Nutrients.

[11] Aral K, Berdeli E, Aral CA, Berdeli A, Atan M (2017). Effects of bodybuilding and protein supplements in saliva, gingival crevicular fluid, and serum. J Oral Sci.

[12] Pullishery F, Dada A, Abdelrasoul MR, Shalaby MA (2020). Periodontal status of 33-44-year-old male bodybuilders and its relationship with protein supplement intake: an observational comparative study. Adv Hum Biol.

[13] Adegboye AR, Boucher BJ, Kongstad J, Fiehn NE, Christensen LB, Heitmann BL (2016). Calcium, vitamin D, casein and whey protein intakes and periodontitis among Danish adults. Public Health Nutr.

[14] Lee J, Park JC, Jung UW, Choi SH, Cho KS, Park YK, Kim CS (2014). Improvement in periodontal healing after periodontal surgery supported by nutritional supplement drinks. J Periodontal Implant Sci.

[15] Abreu OJ, Tatakis DN, Elias-Boneta AR, López Del Valle L, Hernandez R, Pousa MS, Palacios C (2016). Low vitamin D status strongly associated with periodontitis in Puerto Rican adults. BMC Oral Health.

[16] Isola G, Alibrandi A, Rapisarda E, Matarese G, Williams RC, Leonardi R (2020). Association of vitamin D in patients with periodontitis: a cross-sectional study. J Periodontal Res.

[17] Dhulipalla R, Sowjanya CL, Kolaparthy L, Boyapati R, Adurty C, Marella Y (2023). Estimation of serum 1,25-dihydroxycholecalciferol and tumor necrosis factor-α levels in chronic periodontitis. Cureus.

[18] Meghil MM, Hutchens L, Raed A (2019). The influence of vitamin D supplementation on local and systemic inflammatory markers in periodontitis patients: A pilot study. Oral Dis.

[19] Perić M, Maiter D, Cavalier E, Lasserre JF, Toma S (2020). The effects of 6-month vitamin D supplementation during the non-surgical treatment of periodontitis in vitamin-D-deficient patients: a randomized double-blind placebo-controlled study. Nutrients.

[20] Hans M, Malik PK, Hans VM, Chug A, Kumar M (2023). Serum levels of various vitamins in periodontal health and disease: a cross sectional study. J Oral Biol Craniofac Res.

[21] Behfarnia P, Dadmehr M, Hosseini SN, Mirghaderi SA (2021). The effect of vitamin E supplementation on treatment of chronic periodontitis. Dent Res J (Isfahan).

[22] Assaf M, Rabi H (2022). Assessment of vitamin C levels in periodontal patients: a cross-sectional study in Palestine. J Pharm Bioallied Sci.

[23] Munday MR, Rodricks R, Fitzpatrick M, Flood VM, Gunton JE (2020). A pilot study examining vitamin C levels in periodontal patients. Nutrients.

[24] Ghasemi S, Torab Z, Shirmohammadi A (2022). Evaluation of the effect of coenzyme Q10 supplementation along with scaling and root planing (SRP) on periodontal and gingival indices in controlled diabetic patients. J Adv Periodontol Implant Dent.

[25] Shoukheba M, El-Kholy S (2019). Coenzyme Q10 food supplement on the treatment of chronic periodontitis in patients with type II diabetes mellitus: a randomized control study. Egyptian Dental Journal.

[26] Balaji TM, Vasanthi HR, Rao SR (2015). Gingival, plasma and salivary levels of melatonin in periodontally healthy individuals and chronic periodontitis patients: a pilot study. J Clin Diagn Res.

[27] Ghallab NA, Hamdy E, Shaker OG (2016). Malondialdehyde, superoxide dismutase and melatonin levels in gingival crevicular fluid of aggressive and chronic periodontitis patients. Aust Dent J.

[28] El-Sharkawy H, Elmeadawy S, Elshinnawi U, Anees M (2019). Is dietary melatonin supplementation a viable adjunctive therapy for chronic periodontitis?-A randomized controlled clinical trial. J Periodontal Res.

[29] Anton DM, Martu MA, Maris M (2021). Study on the effects of melatonin on glycemic control and periodontal parameters in patients with type II diabetes mellitus and periodontal disease. Medicina (Kaunas).

[30] Zare Javid A, Hosseini SA, Gholinezhad H, Moradi L, Haghighi-Zadeh MH, Bazyar H (2020). Antioxidant and anti-inflammatory properties of melatonin in patients with type 2 diabetes mellitus with periodontal disease under non-surgical periodontal therapy: a double-blind, placebo-controlled trial. Diabetes Metab Syndr Obes.

[31] Invernici MM, Salvador SL, Silva PH (2018). Effects of Bifidobacterium probiotic on the treatment of chronic periodontitis: a randomized clinical trial. J Clin Periodontol.

[32] Ghazal M, Ahmed S, Farooqui WA (2023). A placebo-controlled randomized clinical trial of antibiotics versus probiotics as an adjuvant to nonsurgical periodontal treatment among smokers with Stage III, Grade C generalized periodontitis. Clin Adv Periodontics.

[33] Szkaradkiewicz AK, Stopa J, Karpiński TM (2014). Effect of oral administration involving a probiotic strain of Lactobacillus reuteri on pro-inflammatory cytokine response in patients with chronic periodontitis. Arch Immunol Ther Exp (Warsz).

[34] Riccia DN, Bizzini F, Perilli MG, Polimeni A, Trinchieri V, Amicosante G, Cifone MG (2007). Anti-inflammatory effects of Lactobacillus brevis (CD2) on periodontal disease. Oral diseases.

[35] Wiktorowska-Owczarek A, Berezińska M, Nowak JZ (2015). PUFAs: structures, metabolism and functions. Adv Clin Exp Med.

[36] Castro Dos Santos NC, Furukawa MV, Oliveira-Cardoso I, Cortelli JR, Feres M, Van Dyke T, Rovai ES (2022). Does the use of omega-3 fatty acids as an adjunct to non-surgical periodontal therapy provide additional benefits in the treatment of periodontitis? A systematic review and meta-analysis. J Periodontal Res.

[37] Rautiainen S, Manson JE, Lichtenstein AH, Sesso HD (2016). Dietary supplements and disease prevention - a global overview. Nat Rev Endocrinol.

[38] Caritá AC, Fonseca-Santos B, Shultz JD, Michniak-Kohn B, Chorilli M, Leonardi GR (2020). Vitamin C: one compound, several uses. Advances for delivery, efficiency and stability. Nanomedicine.

[39] Doseděl M, Jirkovský E, Macáková K (2021). Vitamin C-sources, physiological role, kinetics, deficiency, use, toxicity, and determination. Nutrients.

[40] Ashino H, Shimamura M, Nakajima H (2003). Novel function of ascorbic acid as an angiostatic factor. Angiogenesis.

[41] Arenas-Jal M, Suñé-Negre JM, García-Montoya E (2020). Coenzyme Q10 supplementation: efficacy, safety, and formulation challenges. Compr Rev Food Sci Food Saf.

[42] Chan YH, Ho KN, Lee YC (2022). Melatonin enhances osteogenic differentiation of dental pulp mesenchymal stem cells by regulating MAPK pathways and promotes the efficiency of bone regeneration in calvarial bone defects. Stem Cell Res Ther.

[43] Balaji TM, Varadarajan S, Jagannathan R (2022). Melatonin levels in periodontitis vs. the healthy state: a systematic review and meta-analysis. Oral Dis.

[44] Gupta V, Garg R (2009). Probiotics. Indian J Med Microbiol.

